# Effect of *Vitis vinifera* hydroalcoholic extract against oxaliplatin neurotoxicity: *in vitro* and *in vivo* evidence

**DOI:** 10.1038/s41598-018-32691-w

**Published:** 2018-09-25

**Authors:** Laura Micheli, Luisa Mattoli, Anna Maidecchi, Alessandra Pacini, Carla Ghelardini, Lorenzo Di Cesare Mannelli

**Affiliations:** 1 Department of Neuroscience, Psychology, Drug Research and Child Health - NEUROFARBA - Pharmacology and Toxicology Section, University of Florence, Florence, Viale Gaetano Pieraccini 6, 50139 Italy; 2 Aboca S.p.A. Società Agricola, Località Aboca, Sansepolcro, Arezzo, 52100 Italy; 3 Department of Experimental and Clinical Medicine, Anatomy and Histology Section, University of Florence, Florence, Largo Brambilla 1, 50134 Italy

## Abstract

Oxaliplatin treatment is associated with the development of a dose-limiting painful neuropathy impairing patient’s quality of life. Since oxidative unbalance is a relevant mechanism of oxaliplatin neurotoxicity, we assessed the potential antioxidant properties of *Vitis vinifera* extract in reducing oxaliplatin-induced neuropathy as a valuable therapeutic opportunity. A hydroalcoholic extract of *Vitis vinifera* red leaf was characterized and tested in primary rat astrocyte cells treated with oxaliplatin (100 μM). Oxaliplatin lethality in the human adenocarcinoma cell line HT-29 was evaluated in the absence and presence of the extract. *In vivo*, pain hypersensitivity was measured in a rat model of neuropathy induced by oxaliplatin and *ex vivo* molecular targets of redox balance were studied. *Vitis vinifera* extract (50 μg mL^−1^, 4 h incubation) significantly reduced the oxaliplatin-dependent superoxide anion increase and lipid peroxidation in rat astrocytes but did not interfere with the mortality elicited by oxaliplatin in HT-29 cancer cells. In oxaliplatin-treated rats, a repeated daily administration of the *Vitis vinifera* extract (300 mg kg^−1^, p.o.) significantly prevented mechanical and thermal hypersensitivity to noxious and non noxious stimuli. mRNA and protein levels of Nrf2 were normalized in spinal cord and DRGs. Moreover, in the spinal cord, the extract significantly decreased the activation of astrocytes. *Vitis vinifera* reduced oxidative damages and relieved pain without influencing oxaliplatin anti-cancer activity.

## Introduction

Oxaliplatin, a third-generation platinum-based anticancer agent, is mainly used in the treatment of colorectal cancer^1^. It generates the cross-link DNA that leads to the replication failure of cancer cells and has improved cancer therapy^2,3^. However, chemotherapy-induced neuropathy is a common, dose-dependent adverse effect of oxaliplatin. It can lead to detrimental dose reduction and discontinuation of therapy, and severely affects the quality of life of cancer survivors^4^. Currently, the 2014 practical clinical guideline from the American Society of Clinical Oncology states that there are no agents recommended for the prevention of chemotherapy-induced neuropathic pain^5^. Consequently, the prevention or treatment of oxaliplatin-induced neuropathy (as well as other chemotherapy induced neuropathies) is a relevant therapeutic need.

Various studies have reported that natural products could be a great source of new therapeutic compounds essential in treatment of disorder related to pain^6–8^. Natural products have been an important source of substances with relevant therapeutic potential, their efficacy is based on the combined action of a mixture of constituents able to offer a multiple approach to the multi-factorial nature of neuropathy pathogenesis. The bioactivity of crude drugs or vegetal extracts is a summation of antagonistic and/or synergistic effects on bioavailability, cellular transport processes, compound metabolism and pharmacodynamic mechanisms^9^.

*Vitis vinifera* L. (commonly grape) has been used as a food and a beverage, as well as a remedy against various complaints in traditional medicine worldwide since ancient times. Leaves of the plants have been used to stop bleeding and to treat inflammatory disorders and pain^10^. Fresh leaves are also used externally as a folk remedy to heal wounds, to lance abscess^11^ and to reduced blood glucose levels in diabetics. The chemical composition and biological activities of the fruit, seeds and leaves of grape have been extensively investigated^10,12–14^. The leaves are rich in tannins, flavonoids, procyanidins and also contain organic acids, lipids, enzymes and vitamins^10,15,16^. Although chemical composition of *Vitis vinifera* leaves is known very well, the studies conducted on the biological effects of the leaves are limited.

In this study, the protective properties of an hydroalcoholic extract of *Vitis vinifera* red leaf has been tested in oxaliplatin-treated nervous cells. Moreover, the influence of this extract on the cancer cell lethality induced by oxaliplatin has been investigated in the human HT-29 cell line. Furthermore, the anti neuropathic properties of daily administration of the extract have been evaluated in rats subchronically treated with the anticancer drug. At last, molecular targets of redox balance were studied in the nervous system by western blot and PCR.

## Results

**Figure 1:**
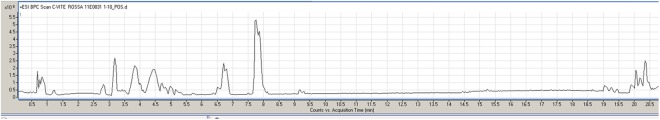
High resolution mass spectrometry chromatographic fingerprint profile of *Vitis Vinifera* leaf freeze dried extract.

According to the liquid chromatography- mass spectrometry (LC-MS) method developed by means of a UHPLC-qToF the *Vitis Vinifera* leaf freeze dried extract metabolomic profile reported in the Fig. 1 has been collected.

The “tridimensional” chromatogram shows all the molecular species presents at the various retention times and their abundances. The chromatogram recorded with a high-resolution mass spectrometer produce a highly specific profile, suitable for identification purpose during routine quality control.

High-resolution masses and corresponding fragmentation pattern of all the species present in the chromatogram (Fig. 1) were compared with “*ad hoc”* natural compounds database and, focusing on anthocyanosides, the structure reported in Table 1 was identified in agreement to literature. For all the reported structures an overall score upper then 50% was found; A mass difference between calculated mass and the experimental value lower than 2 ppm was recorded.

**Table 1:** Anthocyanosides found in *Vitis Vinifera* red leaf freeze dried extract.

Components	%
Phenols, total (as Gallic acid)	22.1
Flavonoids anthocyanins total (as Delphinidin chloride)	1.4
Hydro-soluble Polysaccharides MW > 20000 Dalton	1.32

^a^Mass Difference between calculated mass or exact mass and the experimental value.

^b^The reported value is the overall score to which contribute: the mass score, the isotope abundance score, the isotope spacing score and the retention time score.

In Table 2 was reported more specifically the subclasses of phenols and polysaccharides presented in the extract.

**Table 2:** Composition’s study of *Vitis vinifera* red leaf freeze dried extract.

Compound	Retention time (min)	Accurate m/z	Delta (ppm)^a^	SCORE %^b^	Fragments
Delphinidin-3-O-galactoside	3.53	*465.1035*	*1.51*	93.74	**303.0513**, 84.9601
Kuromanin (cyanidin-3-O-glucoside)	3.87	*449.1086*	*1.78*	89.6	**287.0560**
Oenin (malvidin-3-O-glucoside)	4.55	*493.1345*	*0.81*	95.11	**301.0713**
Peonidin-3-O-glucoside	4.44	*463.1243*	*1.73*	50.83	**301.0714**

### Primary rat astrocytes

**Figure 2:**
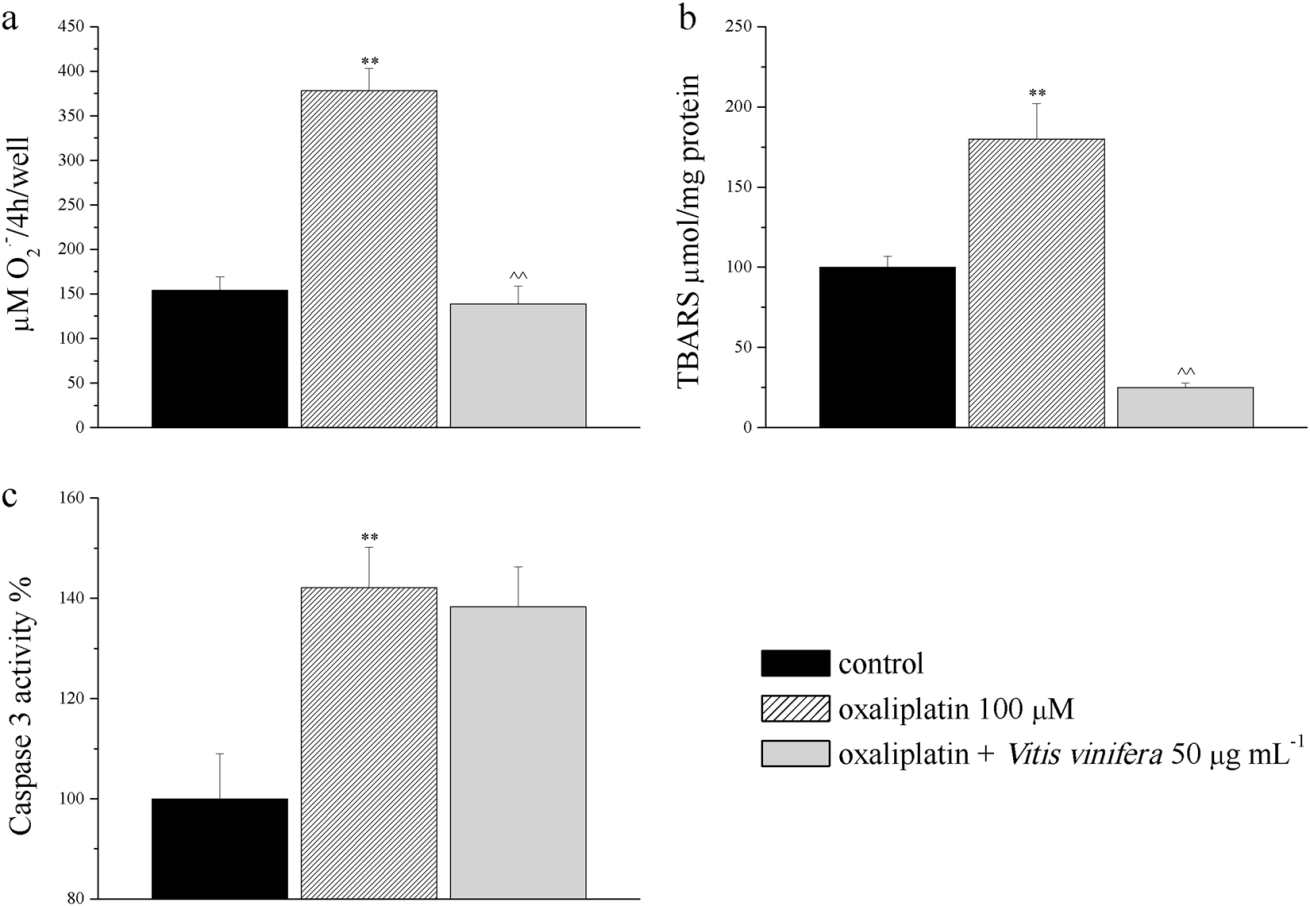
Effect of *Vitis vinifera* extract against damages induced by oxaliplatin in a primary culture of astrocytes. Astrocyte cells were exposed to 100 μM oxaliplatin for 4 h in the presence or absence of 50 μg ml^−1^ *Vitis vinifera* extract. Effect of *Vitis vinifera* extract on SOD-inhibitable O_2_^·−^ concentration (**a**), on TBARS (thiobarbituric acid reactive substances) levels obtained by Fenton reaction (**b**) and caspase 3 activity at 4 h (**c**). **P < 0.01 vs vehicle + vehicle; ^^P < 0.01 vs oxaliplatin + vehicle.

According to our previous results, at least a component of oxaliplatin toxicity is dependent on ROS generation. Since astrocytes are strongly involved in the neuropathic mechanisms of the chemotherapeutic agent^17^, the antioxidant effect of *Vitis vinifera* extract was evaluated in a rat primary culture of these cells. Oxaliplatin (100 µM, 4 h incubation) significantly increased O_2_^−·^ production from 154.0 ± 13.1 μM/4 h/mg (control level) to 380.1 ± 11.4 μM/4 h/mg proteins (Fig. 2a). This increase was totally prevented by 50 μg ml^−1^ *Vitis vinifera* extract (145.4 ± 11.4 μM/4 h/ mg proteins, Fig. 2a). Moreover, the oxidative damage at the lipid component was evaluated after 4 h of 100 μM oxaliplatin treatment. The anticancer drug significantly increased the basal level of TBARS from the value of 100.0 ± 7 μmol/mg to 180.0 ± 22 μmol/ mg proteins, index of lipid peroxidation. Fifty μg ml^−1^ *Vitis vinifera* extract significantly prevented lipid peroxidation (25.0 ± 3.0 μmol/mg proteins; Fig. 2b). At the same concentration, oxaliplatin significantly increased caspase 3 activity at 4 h but the treatment with 50 μg ml^−1^ *Vitis vinifera* was ineffective (Fig. 2c).

### HT-29 cell line

Aimed to evaluate a possible interaction between the treatment with *Vitis vinifera* extract and the therapeutic properties of oxaliplatin, we investigated the vitality of the human colorectal cancer cell line HT-29. Table 3 shows oxaliplatin lethal effect after 24 h incubation (0.1–100 µM) in the absence and presence of the vegetal extract. Fifty µg ml^−1^ *Vitis vinifera* extract did not significantly modified oxaliplatin lethality. The oxaliplatin toxic activity on HT-29 cancer cells was not modified in the presence of the same treatment also after 48 h incubation (Table 4).

**Table 3:** HT-29 cell viability after 24 h incubation.

Oxaliplatin concentration (μM)	Cell Viability % 24 h Incubation	
	Control	*Vitis vinifera* 50 μg mL^−1^
0	100.0 ± 2.6	100.0 ± 2.6
0.3	87.5 ± 7.3	91.0 ± 1.6
1	84.0 ± 5.5	90.5 ± 2.5
3	84.0 ± 2.9	87.4 ± 2.2
10	78.7 ± 3.5	84.6 ± 0.5
30	73.9 ± 3.4	75.1 ± 2.0
100	62.4 ± 2.5	62.7 ± 1.1

HT-29 cells (cells/well) were treated with increasing concentrations of oxaliplatin (1–100 μM) in the presence or absence of 50 μg/mL *Vitis vinifera* extract. Incubation was allowed for 24 h. Cell viability was measured by the MTT assay. The control condition was arbitrarily set as 100% and values are expressed as the mean ± s.e.m. of six experiments.

**Table 4:** HT-29 cell viability after 48 h incubation.

Oxaliplatin concentration (μM)	Cell Viability % 48 h Incubation	
	Control	*Vitis vinifera* 50 μg mL^−1^
0	100.0 ± 1.5	100.0 ± 1.5
0.3	89.1 ± 2.7	92.1 ± 3.0
1	79.2 ± 3.8	89.2 ± 6.4
3	76.2 ± 4.3	81.9 ± 2.5
10	74.3 ± 3.2	80.4 ± 3.6
30	67.7 ± 4.2	69.0 ± 2.6
100	44.0 ± 1.7	43.2 ± 2.0

HT-29 cells (cells/well) were treated with increasing concentrations of oxaliplatin (1–100 μM) in the presence or absence of 50 μg/mL *Vitis vinifera* extract. Incubation was allowed for 48 h. Cell viability was measured by the MTT assay. The control condition was arbitrarily set as 100% and values are expressed as the mean ± s.e.m. of six experiments.

### Behavioral measurements

**Figure 3:**
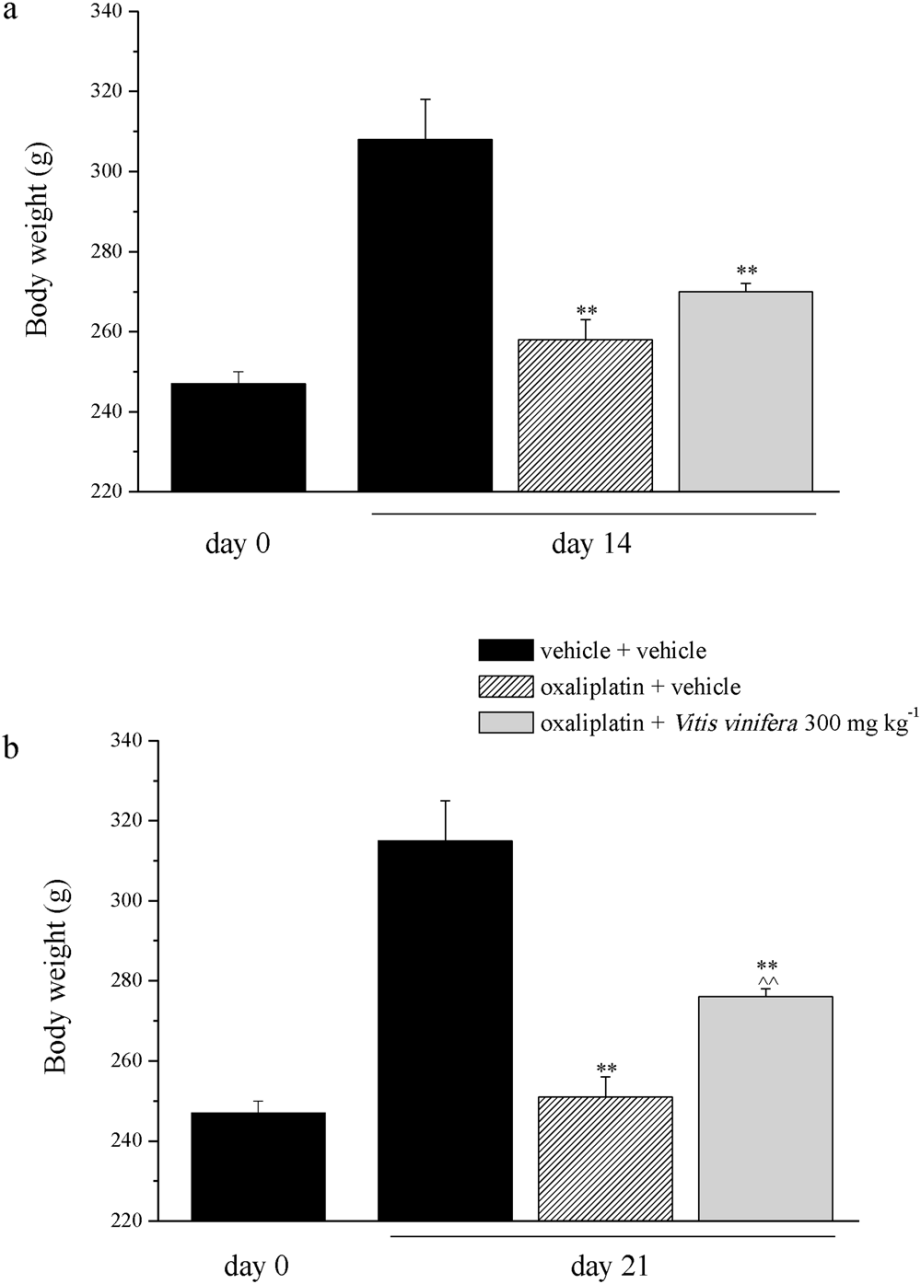
Rat body weight gain with oxaliplatin and *Vitis vinifera* treatments. Animals were treated daily i.p. with 2.4 mg kg^−1^ of oxaliplatin. *Vitis vinifera* extract (300 mg kg^−1^) was daily *per os* administered starting from the first day of oxaliplatin injection. Body weight was measured on day 0, 14 and 21 after the beginning of the experiment. Each value represents the mean ± s.e.m. of 10 rats per group, performed in 2 different experimental sets carried out by experimenters blinded to the treatments. **P < 0.01 vs vehicle + vehicle; ^^P < 0.01 vs oxaliplatin + vehicle.

Intraperitoneally daily administration of oxaliplatin (2.4 mg kg^−1^) significantly lowered the weight gain of the animals following 14 and 21 days (258.2 ± 5.0 g and 251.0 ± 4.6 g, respectively) in comparison to the control group (308.1 ± 10.3 g and 315.0 ± 10.0 g, respectively) (Fig. 3). Daily treatment with *Vitis vinifera* (300 mg kg^−1^, p.o.) significantly reduced the loss of weight observed with oxaliplatin injections on day 21 (Fig. 3b). No significant effect was observed after fourteen days of repeated treatment (Fig. 3a).

**Figure 4:**
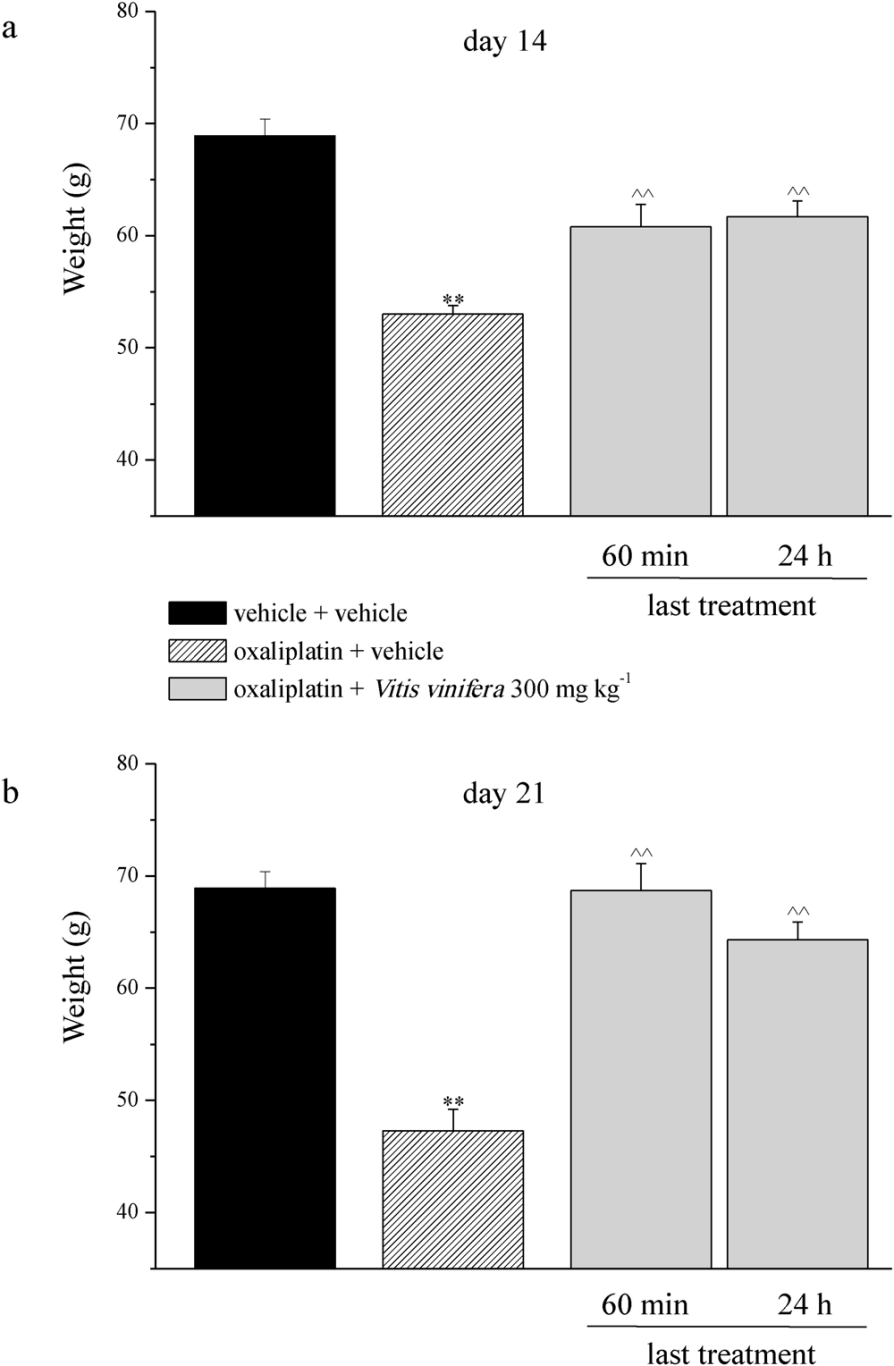
Behavioral measurements. Pain: noxious stimulus. The Paw pressure test was used to measure the sensitivity to a mechanical stimulus. Animals were treated daily i.p. with 2.4 mg kg^−1^ of oxaliplatin. *Vitis vinifera* extract (300 mg kg^−1^) was daily *per os* administered starting from the first day of oxaliplatin injection and behavioral evaluations were performed after 14 and 21 days of treatment, 60 min and 24 h after *Vitis vinifera* administration. Control animals were treated with vehicles. Each value represents the mean ± s.e.m. of 10 rats per group, performed in 2 different experimental sets carried out by experimenters blinded to the treatments. **P < 0.01 vs vehicle + vehicle; ^^P < 0.01 vs oxaliplatin + vehicle.

**Figure 5:**
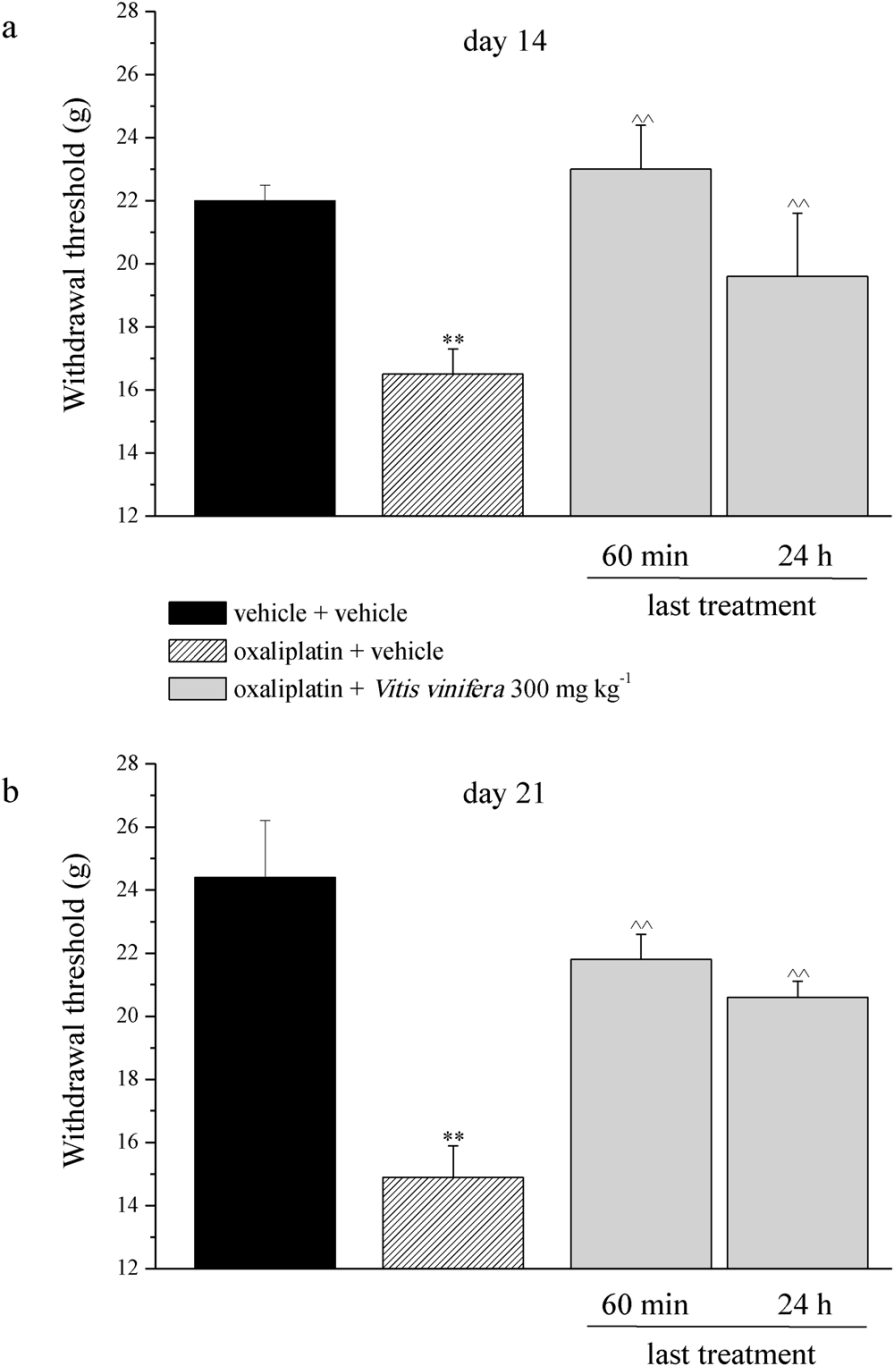
Behavioral measurements. Pain: non noxious stimulus. The Von Frey test was used to measure the pain threshold as a response evoked by a mechanical stimulus. Animals were treated daily i.p. with 2.4 mg kg^−1^ of oxaliplatin. *Vitis vinifera* extract (300 mg kg^−1^) was daily *per os* administered starting from the first day of oxaliplatin injection and behavioral evaluations were performed after 14 and 21 days of treatment, 60 min and 24 h after *Vitis vinifera* administration. Control animals were treated with vehicles. Each value represents the mean ± s.e.m. of 10 rats per group, performed in 2 different experimental sets carried out by experimenters blinded to the treatments. **P < 0.01 vs vehicle + vehicle; ^^P < 0.01 vs oxaliplatin + vehicle.

**Figure 6:**
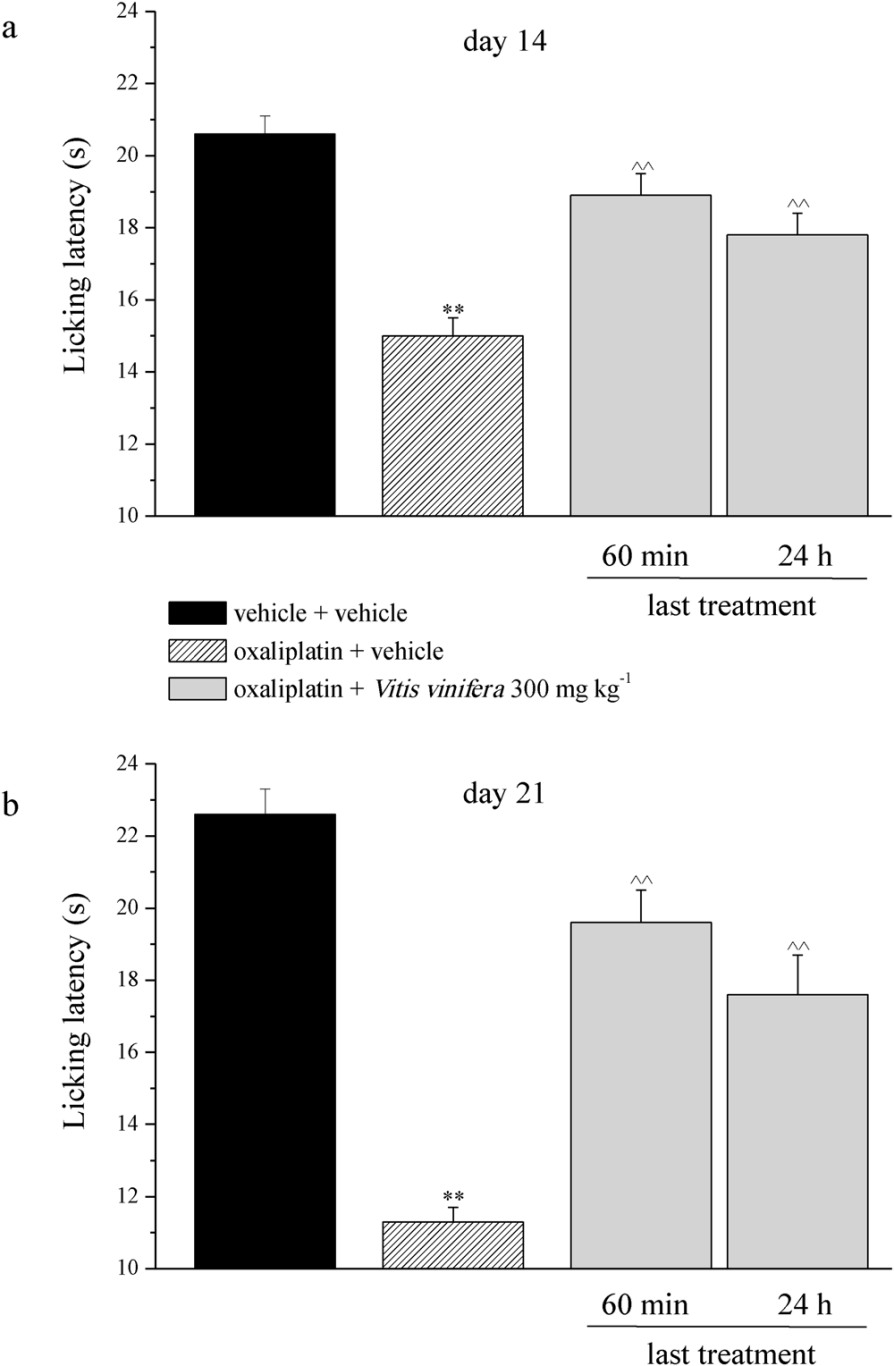
Behavioral measurements. Pain: non noxious stimulus. The response to a thermal stimulus was evaluated by the Cold plate test measuring the latency (s) to pain-related behaviors (lifting or licking of the paw). Animals were treated daily i.p. with 2.4 mg kg^−1^ of oxaliplatin. *Vitis vinifera* extract (300 mg kg^−1^) was daily *per os* administered starting from the first day of oxaliplatin injection and behavioral evaluations were performed after 14 and 21 days of treatment, 60 min and 24 h after *Vitis vinifera* administration. Control animals were treated with vehicles. Each value represents the mean ± s.e.m. of 10 rats per group, performed in 2 different experimental sets carried out by experimenters blinded to the treatments. **P < 0.01 vs vehicle + vehicle; ^^P < 0.01 vs oxaliplatin + vehicle.

Moreover, oxaliplatin daily treatment induced alterations of the pain threshold measured as a response to a noxious mechanical stimulus by the Paw pressure test (Fig. 4). The weight tolerated on the posterior paw progressively decreased from the control value (vehicle + vehicle) of about 70 g to 53.0 ± 0.8 g on day 14 and to 47.3 ± 1.9 g on day 21. Daily *per os* (p.o.) administration of *Vitis vinifera* (300 mg kg^−1^) progressively reduced oxaliplatin-induced hypersensitivity and fully prevented it on day 21, 24 h after the last administration (64.3 ± 1.6 g). The pain reliever effect of *Vitis vinifera* did not significantly improve 60 min after a new administration neither on day 14 nor on day 21 (Fig. 4). Von Frey and Cold plate tests allowed the evaluation of sensitivity to stimuli which normally do not provoke pain. In Fig. 4 the withdrawal threshold (g) to non-noxious mechanical stimulus was reported. Von Frey test highlighted a decreased of pain threshold that started from the 1^st^ week of oxaliplatin treatment (16.5 ± 0.8 g of oxaliplatin + vehicle group *vs* 22.0 ± 0.5 g of vehicle + vehicle group, Fig. 5a) and peaked after 2 weeks (14.9 ± 1.0 g of oxaliplatin + vehicle group *vs* 24.4 ± 1.8 g of vehicle + vehicle group, Fig. 5b). *Vitis vinifera* repeated treatment significantly increased the pain threshold both on days 14 and 21, 24 h the last administration. The efficacy of the vegetal extract did not improved 60 min after a new administration (Fig. 5). The sensitivity to a cold non-noxious stimulus is depicted in Fig. 6. With oxaliplatin treatment the licking latency decreased from about 23 s (vehicle + vehicle) to 15.0 ± 0.5 s on day 14 (Fig. 6a) and to 11.03 ± 0.4 s on day 21 (Fig. 6b). At the observed time points *Vitis vinifera* reduced pain threshold alteration reaching the values of 17.8 ± 0.6 s on day 14 and 17.6 ± 1.1 s on day 21, both recorded 24 h after the last administration of the vegetal extract (Fig. 6). As described previously, a new administration of *Vitis vinifera* did not improved significantly the anti-allodynic properties of the treatment.

### Nuclear factor (erythroid-derived 2)-like 2 (Nrf2) and NAD(P)H dehydrogenase quinone 1 (NQO1) mRNA levels in the nervous system

**Figure 7:**
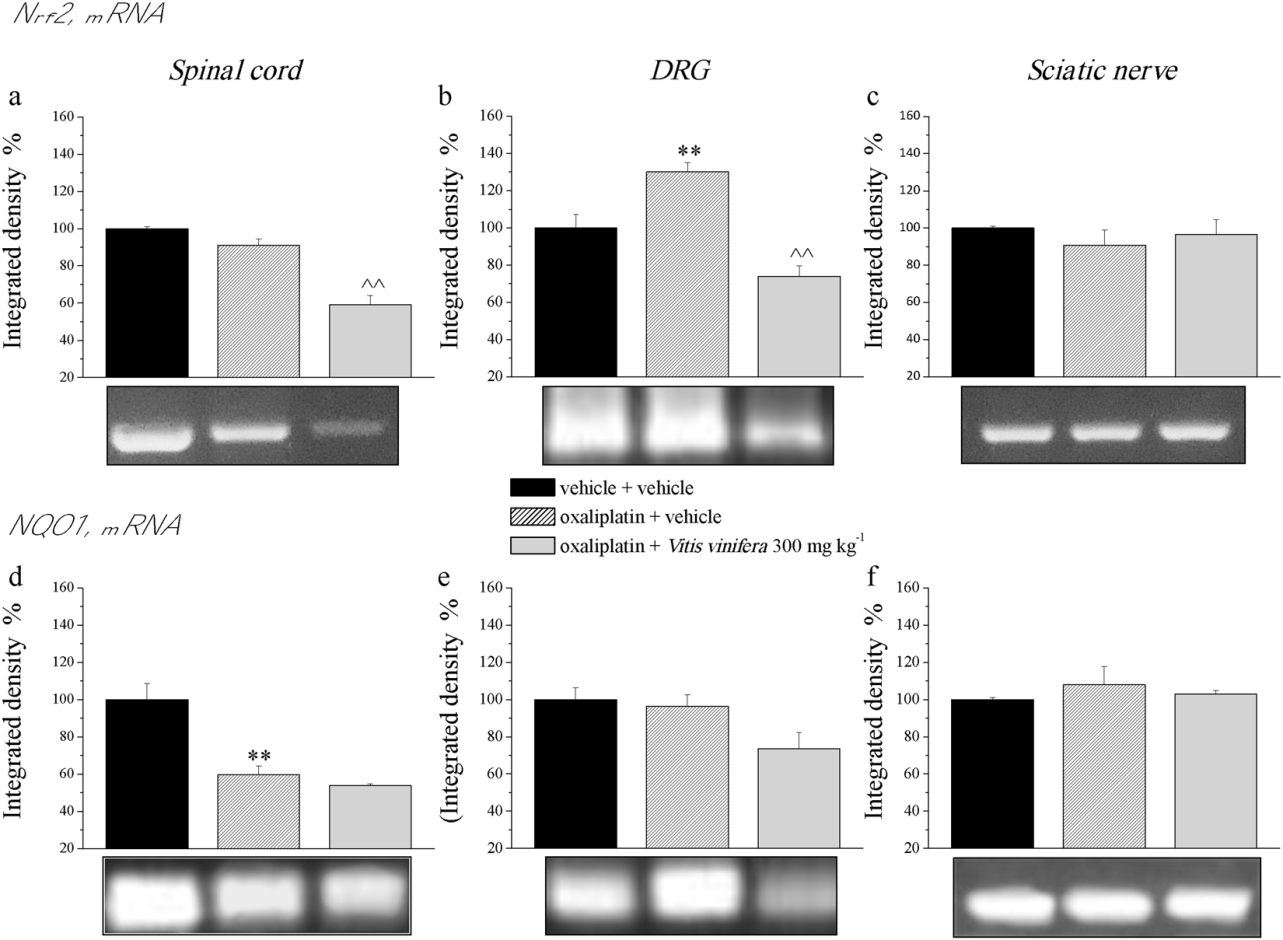
mRNA expression levels of Nrf2 and NQO1 in the nervous system. On day 21, the effect of repeated treatment with *Vitis vinifera* (300 mg kg^−1^ daily p.o.) was evaluated in oxaliplatin-treated rats. mRNA of Nrf2 and NQO1 were evaluated on spinal cord, L4-L5 DRGs and sciatic nerve. Densitometric analysis was performed and normalized versus the expression of the housekeeping 18 S. The integrated density of the control was considered as 100%. Each value represents the mean ± s.e.m. of 10 rats per group, performed in 2 different experimental sets carried out by experimenters blinded to the treatments. **P < 0.01 vs vehicle + vehicle.

Oxaliplatin treatment increased Nrf2 mRNA in the dorsal root ganglia (DRGs) and this alteration was counteracted by *Vitis vinifera* treatment. On the other hand, oxaliplatin did not alter Nrf2 mRNA either in the spinal cord nor in the sciatic nerve (Fig. 7a–c).

Oxaliplatin decreased the NQO1 mRNA level in the spinal cord but no effect was recorded in the DRG and sciatic nerve (Fig. 7d–f).

### Nrf2 protein level in the nervous system

**Figure 8:**
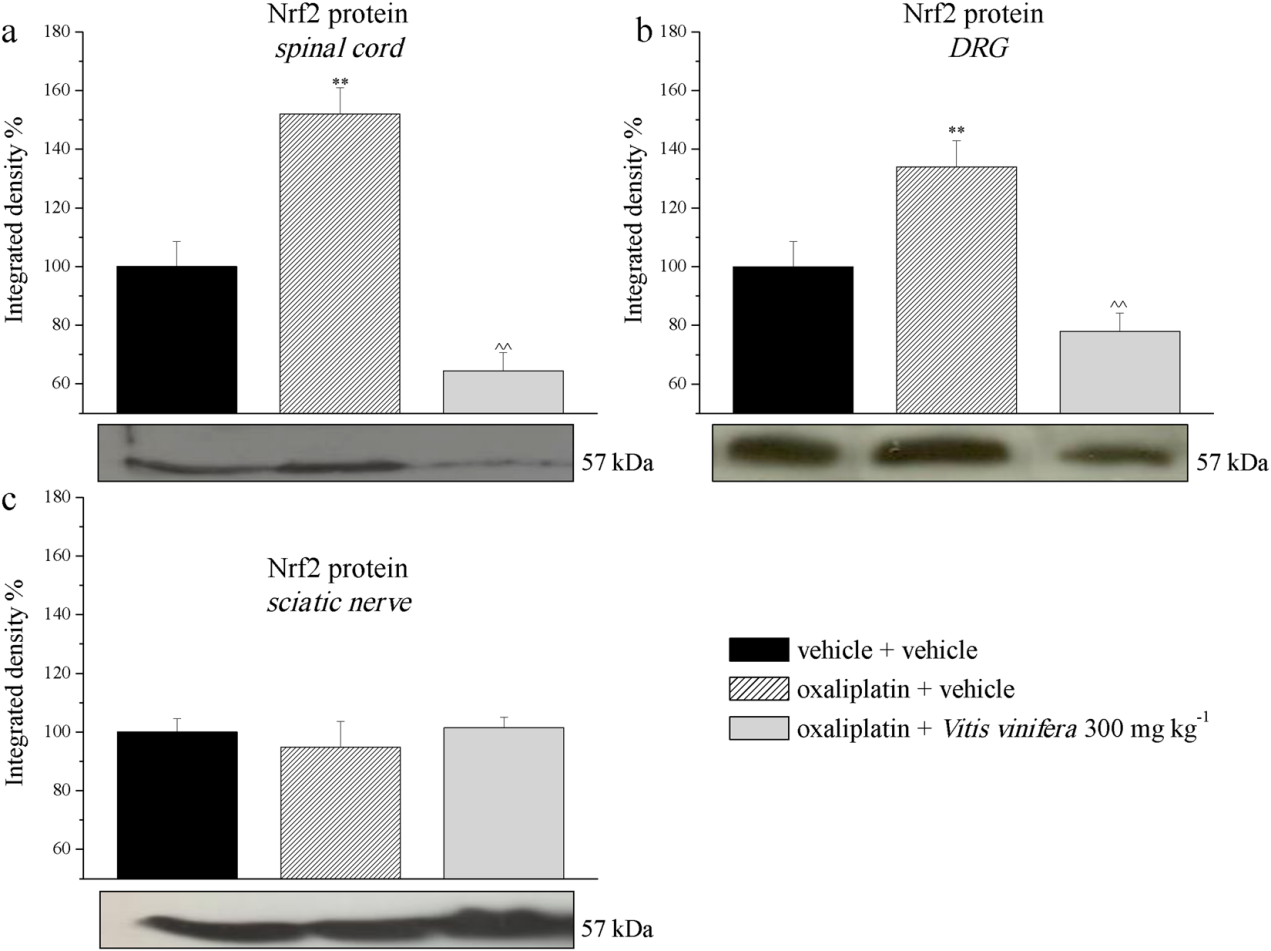
Protein expression levels of Nrf2 in the nervous system. On day 21, the effect of repeated treatment with *Vitis vinifera* (300 mg kg^−1^ daily p.o.) was evaluated in oxaliplatin-treated rats. Western blot of Nrf2 expression in spinal cord, L4-L5, DRGs and sciatic nerve was performed. Densitometric analysis (top) and a representative immunoblot (bottom) are shown. Data are expressed as the percentage of control. β-Actin normalization was performed for each sample. Each value represents the mean ± s.e.m. of 10 rats per group, performed in 2 different experimental sets carried out by experimenters blinded to the treatments. **P < 0.01 vs vehicle + vehicle; ^^P < 0.01 vs oxaliplatin + vehicle.

To assess the antioxidant potential of *Vitis vinifera* after repeated treatment, western blotting was performed to evaluate the expression of Nrf2 in the spinal cord, DRG and sciatic nerve of the rats (Fig. 8). Three weeks of oxaliplatin injections resulted in a significant elevation of Nrf2 levels in the spinal cord and DRG (152.0 ± 8.9 and 134.0 ± 8.5, respectively) in comparison to control animals (Fig. 8a,b). Nrf2 levels were significantly reduced by administration of *Vitis vinifera* in both tissues analyzed (64.5 ± 6.2 and 78.6 ± 6.2, respectively). Oxaliplatin treatment did not modify Nrf2 in the sciatic nerve (Fig. 8c).

### Spinal glial analysis

**Figure 9:**
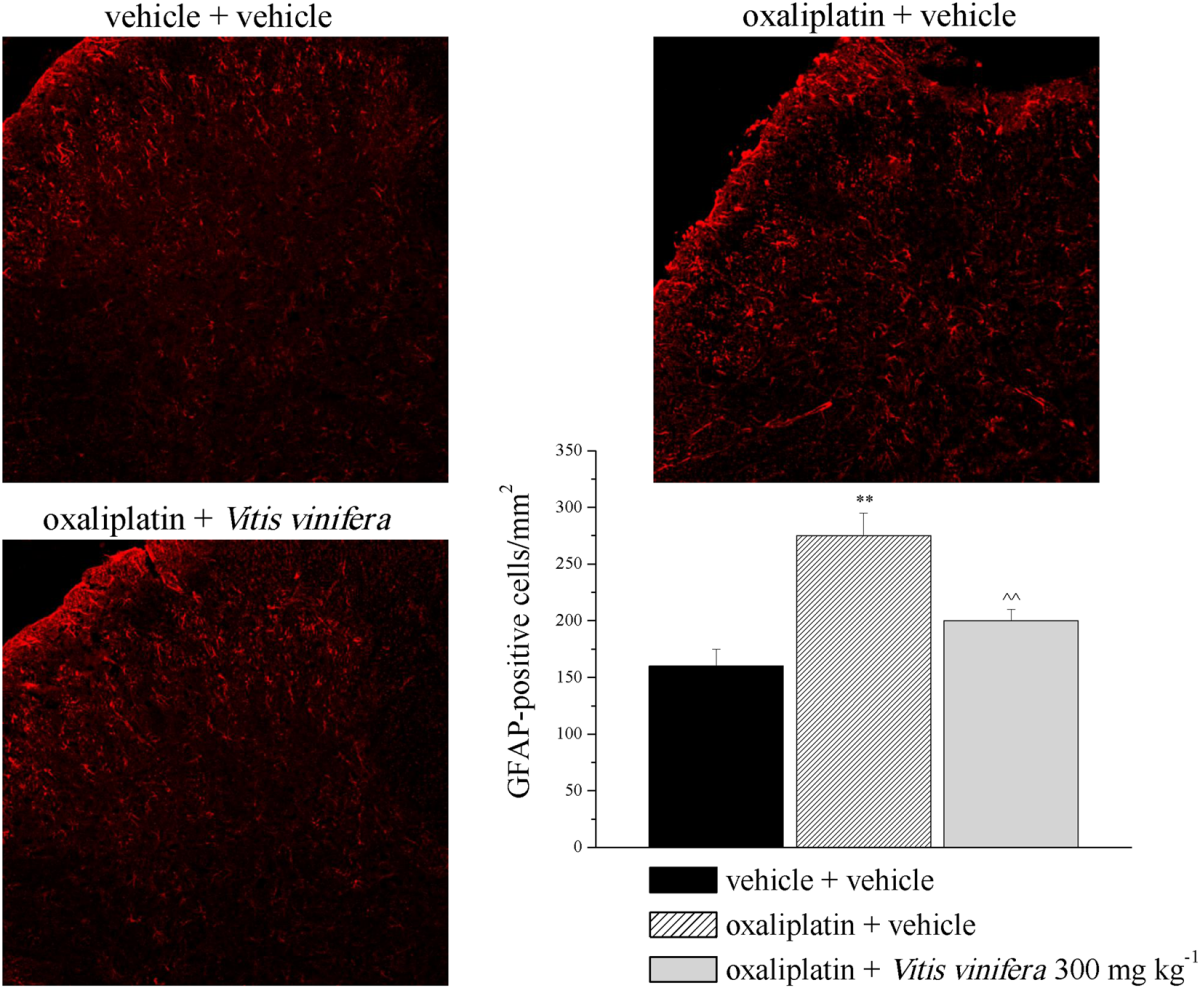
Glial profile in the spinal cord. Effect of repeated treatment with *Vitis vinifera* (300 mg kg^−1^ daily p.o.) was evaluated in oxaliplatin-treated rats. On day 21, the number of GFAP-positive cells was measured in the dorsal horn of the spinal cord. Representative immunohistochemical staining is shown (scale bar = 10 μm and original magnification 20X for all images). Histogram shows quantitative analysis of cellular density. Each value represents the mean ± s.e.m. of 10 rats per group, performed in 2 different experimental sets carried out by experimenters blinded to the treatments. **P < 0.01 vs vehicle + vehicle; ^^P < 0.01 vs oxaliplatin + vehicle.

To establish the relationship between pain relief and glial modulation, the cell density of microglia and astrocytes was measured in the dorsal horn of the spinal cord using immunohistochemistry with antibodies against Iba-1 and GFAP to label microglia and astrocytes, respectively. Oxaliplatin-repeated treatment is linked with the numeric increase of astrocytes but not microglia cells in the spinal cord. Repeated treatment with *Vitis vinifera* extract significantly decreased the number of GFAP-positive cell in the dorsal horn of the spinal cord (Fig. 9). No differences induced by the extract were recorded analyzing the immunohistochemistry for the microglia (Supplementary Figure 1).

## Discussion

The present study shows for the first time the *in vitro* efficacy of a dried extract of *Vitis vinifera* red leaf in reducing cell mortality, oxidative damage and apoptosis induced by oxaliplatin. Moreover, the same extract was tested in a rat model of oxaliplatin-induced neurotoxicity highlighting its anti-neuropathic properties.

The treatment of chemotherapy induced peripheral neuropathy (CIPN) remains largely ineffective. Although different strategies have been attempted, no pharmacological agent has yet been shown to be helpful. As a result, many patients are forced to dose-reduce or discontinue potentially curative neurotoxic drugs the treatment of CIPN remains largely ineffective. Although different strategies have been attempted, no pharmacological agent has yet been shown to be helpful. As a result, many patients are forced to dose-reduce or discontinue potentially curative neurotoxic drugs. The treatment of chemotherapy-induced neuropathy remains largely ineffective. Although different strategies have been attempted, no pharmacological agent has been shown to be completely helpful^18–20^. Despite the mechanisms behind the onset of chemotherapy-induced neuropathy are not clarified yet, Di Cesare Mannelli and colleagues^20^ demonstrated that the oxidative stress has a pivotal role recording this phenomenon at lipid, protein and DNA levels both in plasma as well as in the nervous system^21,22^. *In vitro*, oxaliplatin-dependent nervous alteration may be mimicked using primary astrocytes^19^ measuring oxidative damage and apoptosis induction. Indeed, in physiological conditions, glial cells exert neuroprotective effects by providing neurons with substrates for oxidation^23,24^, but neuropathic dysfunctional glia cells no longer maintain homeostasis and contribute to nervous circuit alterations^23^.

The characterization of our hydroalcoholic *Vitis vinifera* extract highlighted the presence of several bioactive compounds such as phenols, polysaccharides, organic acid (data not shown), vitamins (data not shown) and minerals (data not shown). In particular, the phytochemical characterization revealed that the present extract contained phenol for a total of 22% (expressed as gallic acid) of which anthocyanin a total (expressed as delphinidin chloride) and tannins a total (expressed as pyrogallol) for an amount of 1.4% and 10.2%, respectively. Flavonoids (as the anthocyanins) and tannins, known to exert antioxidant properties^25–28^, could be consider the majors responsible for the extract’s efficacy and mechanism of action, while polysaccharides could be important as natural carrier in the pharmacokinetic behaviour. *Vitis vinifera* hydroalcoholic extract reduced both the presence of oxygen reactive species and the oxidative stress-induced damage to biological molecules after 4 h incubation in astrocytes cell culture. To note, the restorative effect of *Vitis vinifera* did not intervene on the apoptotic process that parallels with the production of O_2_^−·^. Indeed, the extract did not reduce the enhancement of caspase-3 activity, a hallmark of apoptotic cell death^29^. We can hypothesize that *Vitis vinifera* acts as antioxidant agent, reducing ROS production, but not as anti-apoptotic one.

Moreover, *Vitis vinifera* did not interfere with the toxicity elicited by oxaliplatin in the human colon adenocarcinoma cell line HT-29. It is of fundamental importance that, to be clinically useful, the antineuropathic agent must reduce the neurotoxic effect of the chemotherapeutic drug maintaining its full anti-tumor efficacy. We also tested the antineuropathic profile of *Vitis vinifera* subchronic treatment in an animal model of neuropathy induced by oxaliplatin. In clinical, the dosage of oxaliplatin commonly used in human is 85 mg/m^2^ and cumulative doses higher than 1000 mg/m^2^ ^30,31^ induced neuropathic pain in approximately 50% of patients. The human plasmatic concentration of inorganic platinum after a single intravenously administration of 85 mg/m^2^ is about 3 µg/mL and only after repeated cycles (five consecutive cycles at 85 or 130 mg/m^2^ every 3 weeks) a limited accumulation is observed in plasma^32,33^. The animal model followed for the present study is consistent with the clinical practice. The dose of oxaliplatin (2.4 mg kg^−1^) resembles to the common human dosage (considering the Km factor 37 for the conversion of animal doses to the Human Equivalent Dose)^34,35^ and mimics the clinical oxaliplatin cumulative dose leading to chronic neuropathy. *Vitis vinifera* extract was daily per os administered in a dose of 300 mg kg^−1^ starting the same day of oxaliplatin injection. *Vitis vinifera* reduced alterations of the pain threshold evoked by oxaliplatin, in particular when both noxious stimulus (Paw Pressure test) and non-noxious stimuli (Von Frey test and Cold Plate test) were applied.

The anti-neuropathic effect as well as the improvement of body weight was highlighted till 14 days after treatment. Its efficacy was still maintained up to day 21. On both 14 and 21 days, behavioural measurements were performed 24 h and 60 min after the last administration of the extract without record an improvement of its efficacy after 60 min. The fact that the pain relief was observed only after a subchronic treatment suggests that *Vitis vinifera* acts with a neuroprotective mechanism against the damages that results in chronic pain. The antineuropathic properties of an extract of *Vitis vinifera* was previously described by Jin and colleagues (2013)^36^ in a mouse model of diabetic peripheral neuropathy^36^. In that study the efficacy of the vegetal extract was partially due to the oligomeric or polymeric flavonoid-like polyphenolic compounds called proanthocyanidins that have strong antioxidant and free radical-scavenging effects against oxidative damage. Increased levels of reactive oxygen species (ROS) in the body cause cell damage or cell death. Therefore, removal of excessive ROS is important to restore normal conditions. It is a proven fact that ROS are one of the major perpetrators in the induction and progression of neuropathic pain^37^. On the other hand, the mechanism by which oxaliplatin provokes ROS increase is not completely established and it could be due to a characteristic cell damage. A mitochondrial alteration has been suggested as a mechanism of oxaliplatin-mediated oxidation^38^. Moreover, dynamin-related protein 1, a protein that catalyzes the process of mitochondrial fission with the consequent ROS production, is involved in chemotherapy-induced neuropathy in rats^39^.

Since the phytocomplex of *Vitis vinifera* possesses a remarkable activity against oxaliplatin-induced oxidative damages relevant for pain sensitivity^21^, we evaluated in the central and peripheral nervous system protein and mRNA levels of molecules related to redox activity. Nuclear factor erythroid-2 related factor 2 is a key transcription factor and master regulator of the cellular response of oxidative stress, which can induce the expression of antioxidant and detoxification enzymes and downstream proteins such as NAD(P)H: quinone oxidoreductase-1, catalase, superoxide dismutase (SOD), heme oxygenase-1, glutathione peroxidase, and glutathione-S-transferase^40,41^. NQO1 is a cytosolic antioxidant flavoprotein that catalysed the reduction of quinones to hydroquinones by utilizing NADH as an electron donor, which consequently increases intracellular NAD^+^ levels^42,43^. In addition, there is evidence that NQO1 has a role in other biological activities, including anti-inflammatory processes, the scavenging of superoxide anion radicals, and the stabilization of p53^44–48^. Three weeks of oxaliplatin treatment increased Nrf2 protein level in the spinal cord and DRGs while no differences were detected in the sciatic nerve. *Vitis vinifera* was able to counteract this enhancement in both tissue analyzed. Nrf2 mRNA was increased only in the DRGs by oxaliplatin and this effect was reduced by the natural extract. On the other hand, oxaliplatin decreased NQO1 mRNA only in the spinal cord with no effects recorded for *Vitis vinifera* treatment. Moreover, since glial cells have been recognized as powerful modulators of pain, participating in the maladaptive plasticity of the central nervous system, facilitating nociceptive processes and generating clinical pain hypersensitivity^49^, we analysed the effect of *Vitis vinifera* extract on microglia and astrocytes cells. The extract was able to significantly reduced astrocytes activation induced by oxaliplatin treatment without altered the number of microglia cells. Previous experiment showed the relationship existing between oxaliplatin-dependent pain and the increase in glial cell number in the spinal cord^50^. In particular a major role of astroglia in pain perception was highlighted since fluorocitrate, the astrocyte inhibitor, showed a higher efficacy in comparison to minocycline, a microglial inhibitor, to reduce oxaliplatin-induced neuropathy^50^. The inhibitory effect of *Vitis vinifera* on astrocytes can be considered one of the aspects at the base of its neuroprotective and anti-hyperalgesic effect.

## Conclusions

The hydroalcoholic *Vitis vinifera* red leaf extract was able to protect against oxaliplatin-induced oxidative damage and neuropathy in *in vitro* and *in vivo* experiments and it could be a candidate for the treatment of chemotherapy-induced neuropathy.

## Materials and Methods

### *Vitis Vinifera* red leaf, freeze dried extract production

The production process is characterized by extracting the leaf of *Vitis vinifera* with ethanol 60% (v/v) (D/S 1:8). After 8 h, at room temperature, the hydroalcoholic herb’s mixture was dropped for one hour and filtered to remove the exhausted herb. The resulting clarified extract was concentrated under vacuum to evaporate ethanol and the obtained aqueous concentrate underwent to freeze-drying for 72 hours. The resulting freeze dry extract was stored until use, away from light and moisture. (DER 5-8:1). More details are reported in the supplementary materials.

### Phenols total, expressed as gallic acid

All the operations were conducted maintaining the sample protected from light. The total phenols were expressed as gallic acid. Full description is reported in the supplementary material.

### Anthocyanins total, expressed as delphynidin chloride

The total anthocyanins were expressed as delphynidin chloride. Full description is reported in the supplementary material.

### Hydrosoluble Polysaccharides >20KDa

Hydrosoluble polisaccharides >20KDa were detected. Full description is reported in the supplementary material.

### LC-HRMS Fingerprint by UHPLC ESI-QToF

The grinded sample (0.25 g) is extracted with 25 ml of MeOH 0.1% HCl (vol/vol) by means of ultrasound at 35 °C. After 30 minutes the samples is centrifuged 10 minutes at 4000 rpm. The supernatant is collected in a 50 ml volumetric flask and the pellet was extracted in the same conditions. After centrifugation the second extract was combined to the first one and the volume is made up to volume (50 ml) using the same solvent. The sample was filtered on a 0.20 μm cellulose acetate syringe filter, diluted 1:10 and then used for the acquisition of the chromatographic profile (positive ion mode).

### Cell cultures

The HT-29 human colon cancer cell line were acquired from the American Type Culture Collection. HT-29 were cultured in Complete Medium (CM) composed by high-glucose DMEM with 10% FBS, 2 mM L-glutamine, 100 IU/mL penicillin, and 100 μg mL^−1^ streptomycin (Sigma-Aldrich, Italy). Primary cultures of astrocytes were obtained according to the method described by^51^. Full description is reported in the supplementary material. Astrocytes were starved in serum-free DMEM overnight before all treatments. Bicinchoninic acid (Sigma-Aldrich, Italy) assay was used to measure protein homogenate concentrations.

### Cell viability assay

96-well cell culture plates (Corning) were used to plate HT-29 cells (5 × 10^3^ cells/well). After 48 h the experiments were performed. Cells were incubated in serum-free DMEM with 0.3–100 μM oxaliplatin (Carbosynth, Compton, Berkshire, UK) in the absence or presence of 50 μg mL^−1^ *Vitis vinifera* for 24 h or 48 h. Full description is reported in the supplementary material.

### SOD-inhibitable superoxide anion production evaluated by cytochrome c assay

Astrocytes were plated in 6-well plates (5 × 10^5^/well) and grown until confluence. Cells were then incubated with or without 100 μM oxaliplatin in serum-free DMEM containing cytochrome c (1 mg/mL) for 4 h at 37 °C, in the absence or presence of *Vitis vinifera* 50 μg mL^−1^. Extract concentration was chosen on the base of the literature^52^. Non-specific cytochrome c reduction (following the reaction Fe^3+^ + O2-Fe^2+^ + O_2_) was evaluated carrying out tests in the presence of bovine SOD (Sigma-Aldrich, S9697, 300 mU/mL)^53^. Full description is reported in the supplementary material.

### Lipid peroxidation (thiobarbituric acid-reactive substances)

Primary astrocytes (10^6^ cells/flask) were plated in 25-cm^2^ cell culture flasks (Corning), and the experiments were performed after 48 h. Thiobarbituric acid-reactive substances (TBARS) as an index of lipid peroxidation were quantified after 16 h incubation with 100 μM oxaliplatin in the presence of 50 μg mL^−1^ *Vitis vinifera*. The oxaliplatin incubation was optimized previously^53^. Full description is reported in the supplementary material.

### Caspase-3 activity

6-well cell culture plates (Corning) were used to plate primary astrocytes (3 × 10^5^ cells/well). After 48 h the experiments were performed. The cells were incubated with 100 μM oxaliplatin in the presence of 50 μg mL^−1^ *Vitis vinifera* for 4 h. The oxaliplatin incubation was optimized previously^53^. Full description is reported in the supplementary material.

### Animals

Male Sprague–Dawley rats (Envigo, Varese, Italy) weighing 220–250 g at the beginning of the experiments were used. Animals were used at least one week after their arrival and were housed in CeSAL (Centro Stabulazione Animali da Laboratorio, University of Florence). Per cage (size 26 × 41 cm^2^) were housed four rats, animals were nourished with standard laboratory diet and tap water ad libitum, kept at 23 ± 1 °C with a 12 h light/dark cycle, light at 7 a.m. All animal manipulations were carried out according to the Directive 2010/63/EU of the European parliament and of the European Union council (22 September 2010) on the protection of animals used for scientific purposes. The ethical policy of the University of Florence complies with the Guide for the Care and Use of Laboratory Animals of the US National Institutes of Health (NIH Publication No. 85-23, revised 1996; University of Florence assurance number: A5278-01). Formal approval to conduct the experiments described was obtained from the Italian Ministry of Health (No. 54/2014-B) and from the Animal Subjects Review Board of the University of Florence. Experiments involving animals have been reported according to ARRIVE guidelines^54^. All efforts were made to minimize animal suffering and to reduce the number of animals used.

### Oxaliplatin rat model of neuropathy and *Vitis vinifera* treatment

Oxaliplatin (2.4 mg kg^−1^. Carbosynth, Compton, Berkshire, UK) was dissolved in 5% glucose solution and administered intraperitoneally (i.p.) for 5 consecutive days every week for 3 weeks (15 i.p. injections)^55^. Control animals were treated with an equivalent volume of 5% glucose i.p. (vehicle).

*Vitis vinifera* extract was suspended in 1% carboxymethylcellulose (CMC) and orally administered^56^ (p.o.) at 300 mg kg^−1^ for 5 consecutive days every week for 3 weeks (15 i.p. administrations) starting from the first day of oxaliplatin injection. Behavioral tests were performed on days 14 and 21, 1 h and 24 h after the last administration of the vegetal extract.

### Paw pressure test

The mechanical hyperalgesia in the rat was determined with an analgesimeter (Ugo Basile, Varese, Italy) according to Leighton *et al*.^57^. Full description is reported in the supplementary material.

### Von Frey Test

The measure was performed according to the method described by^58^. Full description is reported in the supplementary materials.

### Cold plate test

The measure was performed according to the method described by^59^. Full description is reported in the supplementary materials.

### Tissue collection

At the end of the behavioural test session on day 21, animals were sacrificed by decapitation. Dorsal root ganglia (DRG) L4-L5 and sciatic nerve were dissected and frozen using liquid nitrogen. After dissection, this lumbar portion was frozen using liquid nitrogen.

### Western Blott analysis

Western blott analysis was performed on day 21 at the end of the behavioural measurements. Full description is reported in the supplementary materials.

### mRNA level analysis

Analysis of mRNA levels was performed on day 21 at the end of the behavioural measurements. Full description is reported in the supplementary materials.

### Immunohistochemistry of spinal cord glia

On day 21, SD rats were sacrificed, the L4/L5 segments of the spinal cord were exposed from the lumbovertebral column via laminectomy and identified by tracing the dorsal roots from their respective DRG. Quantification of the number and morphology of Iba1 immunoreactive microglia (rabbit, 1:1000; Wako Chemicals, Richmond, USA) and GFAP immunoreactive astrocytes (mouse, 1:5000; Chemicon, Temecula, USA) in the superficial dorsal horns of the spinal cord areas were performed in four cryostat sections (20 μm) by a previously reported method^50,60^. For details see the supplementary material.

### Statistical analysis

Group size was based on our previous results^61,62^ to allow for the detection of differences with a sufficient power of 80% at the level of significance of 0.05. Statistical analysis results were expressed as means ± s.e.m. and the analysis of variance was performed by ANOVA. A Bonferroni’s significant difference procedure was used as post-hoc comparison. P values of less than 0.05, 0.01 or 0.001 were considered significant. Data were analyzed using the “Origin 8.1” software.

## Electronic supplementary material

Supplementary material
