# Supplementary material for: Effect of Vitis vinifera hydroalcoholic extract against oxaliplatin neurotoxicity: in vitro and in vivo evidence
Source: Sci Rep. 2018 Sep 25;8:14364. doi: 10.1038/s41598-018-32691-w (PMC6156221; doi:10.1038/s41598-018-32691-w)

Effect of *Vitis vinifera* hydroalcoholic extract against oxaliplatin neurotoxicity: *in vitro* and *in vivo* evidence

Laura Micheli[1], Luisa Mattoli[2], Anna Maidecchi[2], Alessandra Pacini[3], Carla Ghelardini[1], Lorenzo Di Cesare Mannelli[1*]

[1]Dept. of Neuroscience, Psychology, Drug Research and Child Health - NEUROFARBA - Pharmacology and Toxicology Section, University of Florence, Florence, Viale Gaetano Pieraccini 6, 50139, Italy

[2]Aboca S.p.A. Società Agricola, Località Aboca, Sansepolcro, Arezzo, 52100, Italy

[3]Dept. of Experimental and Clinical Medicine, Anatomy and Histology Section, University of Florence, Florence, Largo Brambilla 1, 50134, Italy

[*]Corresponding author: Lorenzo Di Cesare Mannelli, Dept. of Neuroscience, Psychology, Drug Research and Child Health - Neurofarba - Pharmacology and Toxicology Section, University of Florence, Viale Pieraccini 6, 50139, Florence, Italy.

Supplementary

Materials and Methods

Vitis Vinifera red leaf, freeze dried extract production

Ultrahigh purified water used in this study was prepared in a PurelabUltra water purification system (ELGA, UK). Absolute ethanol 99,8% (cod 1.11727), methanol 99% (cod. 1.06035), Folin-Ciocalteu (cod. 1.09001) reagent, Calcium Carbonate (cod 1.02066) and Hydrochloric acid 37% (cod. 1.00314) were purchased from Merk-vwr. Absolute methanol LC-MS grade (cod. 695076), Dextran 25000 Da (cod. 00271), formic acid LC-MS grade (cod. 695076), Gallic acid (cod. 91215), were purchased from Sigma-Aldrich. Delphynidin chloride (cod. 09048) was purchased from Extrasynthese. Spectra/Por 3 Dialysis membrane 3.5kD MWCO (cod. 132724T) was purchased from Spectrum laboratories, Inc.

Phenols and anthocyanins analysis was performed by means of a Cary 60 UV-Vis spectrophotometer (Agilent Technologies). Polysaccharide analysis was performed by means of an HPLC 1260 (Agilent Technologies INC., Santa Clara, CA) system equipped with a vacuum degasser, a binary pump, a Peltier thermostated autosampler at 10°C, a Peltier thermostated column compartment and the effluent was analyzed by a refractive index detector (RID). Fingerprint analysis was performed by means of an UHPLC (Agilent 1290 Infinity II) coupled with *Dual AJS*

ESI source Q-ToF (Agilent 6545). The UHPLC is equipped with a vacuum degasser, a binary pump, a Peltier thermostated autosampler at 10°C, a Peltier thermostated column compartment.

Phenols total, expressed as gallic acid

The grinded sample (0.3 g) is extracted with 100 ml of ethanol 50 % (vol/vol) by means of ultrasound at 35° C. After 30 minutes the samples is centrifuged 10 minutes at 4000 rpm. The supernatant is collected in a 100 ml volumetric flask ad it is made up to volume by ethanol 50 % (vol/vol). The resulting solution (0.5 ml) is introduced in a 100 ml volumetric flask containing ultrapure water (50 ml), then Folin-Ciocalteu reagent (5 ml) and Calcium carbonate 20% solution (10 ml) are added, respectively. The volume is made up to 100 ml by ultrapure water. After two hours the absorbance of the solution is read at 760 nm, using water as reference solution.

Using a gallic acid calibration curve (y= 158.42x+0.0068) with concentration ranging from 0.002 mg/ml to 0.004 mg/ml, the total phenols percentage expressed as gallic acid was determined with the following calculation: % of total phenol (as gallic acid) = [(A-a)*F1/(b*p)], where **A** is the sample absorbance at 760nm; **p** is the sample weight in grams; **a** is the intercept of calibration curve; **b** is the slope of calibration curve; **F1** is the Dilution factor, calculated as follows: Ve*Vc/V*10, where **Ve** is the volume of the initial extract; **Vc** is the volume of the colorimetric reaction; **V** is the volume of the initial extract used for the colorimetric reaction.

Anthocyanins total, expressed as delphynidin chloride

The grinded sample (0.025 g) is extracted with a concentrated hydrochloric acid methanol solution 2% (vol/vol) (25 ml) by means of ultrasound at 35° C. After 30 minutes the samples is centrifuged 10 minutes at 4000 rpm. The supernatant is collected in a 50 ml volumetric flask. The extraction is repeated on the residue in the same condition, then the volume is made up to volume (50 ml) using the same solvent. The resulting solution is diluted 1:25 with the same solvent, after which the absorbance of the sample is read at 540 nm, using concentrated hydrochloric acid methanol solution 2% (vol/vol) as reference solution. Knowing that the delphynidin chloride A1%, 1 cm is 1020, the total anthocyanins percentage expressed as delphynidin chloride is calculated using the following calculation: % of total anthocyanins (as delphynidin chloride)= (A*F*V)/(1020*p), where **A** is the sample absorbance at 540nm; **F** is the Dilution factor, **V** is the volume of the sample solution, **p** is the sample weight in grams.

Hydrosoluble Polysaccharides >20KDa

The grinded sample (1.0 g) is extracted with 25 ml of ultra pure water by means of ultrasound at 35° C. After 30 minutes the samples is centrifuged 10 minutes at 4000 rpm. The supernatant is collected in a 50 ml volumetric flask. The extraction is repeated on the residue in the same condition, then the volume is made up to volume (50 ml) using the same solvent. The sample (5 ml) is dialyzed at room temperature, using a dialysis membrane of 3.5 KDa pore cut-off. After three days the sample retentate is collected and made up to volume (10 ml) with water and used to perform the polysaccharides analysis by a HPLC-RID method according the following conditions: column: PL-aquagel OH 40 (Agilent Technologies Inc Santa Clara, CA, USA, 7.5 x 25x300 mm, 8 μm); column temperature: 20°C; flow rate: 1 ml/min; Volume Injected: 10μL; RID temperature: 45°C, Signal polarity = positive; peak width = >0.1min. (response time = 2.0 s, 2.5 Hz); output zero off set = 5%; attenuation 1000 mAu; Eluent: water 0.02% NaN3 during 20 min; post time:1min. Dextran 25000 Da was used as external standard, that was used after solubilization in a 0.02% NaN3 water solution. The concentration of polysaccharides was calculated by means of a calibration curve between the range of 2-0.12 mg/ml.

LC-HRMS Fingerprint by UHPLC ESI-QToF

Chromatographic conditions: column RP-18 (C_{18} Cortecs, Waters) 1.6 um, 2.1 x 100 mm; Column temperature: 40°C; Flow rate: 0.3 ml/min; Injection volume: 3 uL; Mobile phase composition. Solvent A: Ultrapure water containing 0.1% formic acid, Solvent B: Absolute methanol containing 0.1% formic acid; Elution. 0-1 min A 99%:B 1% isocratic, 1-2 min A 75%:B 25% linear gradient, 2-10 min A 50%:B 50% linear gradient, 10-11 min A 50%:B 50% isocratic, 11-15 min A 25%:B 75% linear gradient, 15-17 min A 15%:B 85% linear gradient, 17-19 min A 1%:B 99% linear gradient, 19-19.5 min A 1%:B 99% isocratic; 19.5-21 min A 99%:B 1% linear gradient; equilibration time 3 min.

Mass instrument parameters. Ion source: Dual AJS ESI; Ion polarity: positive; Data storage: Centroid; Stop time: no limit/as pump; Time segment and experiment. # Time: 0 min in WASTE, Time: 2 min in MS, Expt: 1-4; LC stream: MS; MS Abs threshold: 200 MS Rel; threshold: 0.01 %; Source. Gas Temp: 325 °C; Gas flow: 11 L/min; Nebulizer: 35 psig; Shealth Gas Temp: 350°C; Shealth Gas Flow: 12 L/min; Fragmentor: 100 V; Skimmer: 65 V; OCt 1 RF Vpp: 750 V; VCap: 3500 V; Nozzle voltage: 1000. Acquisition: Data were acquired in MS mode using “low channel” and “high channel” with selected Collision Energy (all ion). Mass range: Min range: 50 m/z; Max range: 1700 m/z ; Acquisition rate time. Rate: 2 spectra/sec; Time: 500 ms/spectrum; Transients/spectrum: 4036; Collision Energy: 0-20-30-40 V. Reference mass. Ref mass correction: enable; Reference masses: 121.0509 m/z, 922.0098 m/z; Detection window: 400 ppm; Minimun

height: 1000 counts; Chromatograms. Chromatogram: TIC; Label: TIC; Expt type: MS; Polarity type: Positive, Offset: 15, Y-range: 10000000.
Software. Data were acquired by means of MassHunter workstation software (Version B.06.01). Elaborations were performed by means of "Qualitative analysis" program and by "Find-by-Formula" algorithm. Database searching: The data file is loaded into "Qualitative analysis" then "Find-by-Formula" is run on the low channel against an MS/MS library. Find-by-Formula returns possible precursor formulas found as well as their product ions in the library. Using the list of product ions, "Qualitative analysis" extracts EICs from the high channel and aligns them with an EIC of the precursor. A coelution score is calculated and compounds which pass the threshold (user-set at 80) are retained. Data Analysis. The high resolution mass spectrometry chromatographic fingerprint profile was obtained (figure 1, BPC, base peak chromatogram). After matching with the Aboca's natural compounds library and using the ion "find by formula" algorithm, focusing on anthocyanosides the molecules found are reported in table 1.

Astrocytes cell culture

Primary cultures of astrocytes were obtained according to the method described by McCarthy and de Vellis[51]. Briefly, the cerebral cortex of newborn (P1–P3) Sprague-Dawley rats (Envigo, Italy) was dissociated in Hanks' balanced salt solution (HBSS) containing 0.5%trypsin/EDTA and 1% DNAse for 30 min at 37 °C. The suspension was mechanically homogenized and filtered. Cells were plated in Dulbecco's Modified Eagle's Medium(DMEM)high glucose with 20% fetal bovine serum (FBS, Gibco, Invitrogen, Italy). Confluent primary glial cultures were used to isolate astrocytes removing microglia and oligodendrocytes by shaking. The purity of astrocyte cultures was determined immunocytochemically by staining for GFAP (Dako, Denmark). Cells were fixed in 4% paraformaldehyde, then incubated with the antibody (1:200) and visualized using Alexafluor conjugated secondary antibody. Nuclei were stained with 4′,6-diamidino-2-phenylindoledihydrochloride (DAPI). GFAP-positive cells were 95–98% in astrocyte cultures. Experiments were performed 21 days after cell isolation. Astrocytes were cultured in DMEM high glucose with 10% FBS, 2 mM L-glutamine, 1% essential aminoacid mix, 100 IU/ml penicillin and 100 μg/ml streptomycinin 5% CO_2 atmosphere at 37 °C.
Astrocytes was starved in serum-free DMEM overnight before all treatments. Protein homogenate concentrations were measured by bicinchoninc acid (BCA; Sigma-Aldrich, Italy) assay.

Cell viability assay

Cell viability was evaluated by the reduction of 3-(4,5-dimethylthiozol-2-yl)-2,5-diphenyltetrazolium bromide (MTT; Sigma-Aldrich, Italy) as an index of mitochondrial compartment functionality. After the 24 or 48 h incubation, 1 mg mL^{-1} MTT in serum-free DMEM without phenol red was added into each well and incubated for 30 minutes at 37 °C. After washing,

the formazan crystals were dissolved in 150 μL dimethyl sulfoxide. The absorbance was measured at 550 nm. Experiments were performed in quadruplicate on at least three different cell batches.

SOD-inhibitable superoxide anion production evaluated by cytochrome c assay

The supernatants were collected, and the optical density was spectrophotometrically measured at 550 nm. After subtracting the non-specific absorbance, the SOD-inhibitable O_2^{-} amount was calculated by using an extinction coefficient of $2.1 \cdot 10^4$ $M^{-1}cm^{-1}$ and expressed as μM/mg proteins/4 hours. The 4h incubation interval was chosen on the basis of preliminary experiments which showed poor reliability for longer cytochrome c exposure to the cellular environment[18].

Lipid peroxidation (thiobarbituric acid-reactive substances)

Thiobarbituric acid-reactive substances (TBARS) assay was assessed as an index of lipid peroxidation. The TBARS determination was carried out in primary astrocytes cell cultures after 16 h incubation with 100 μM oxaliplatin in the presence or in the absence of 50 μg mL^{-1} *Vitis vinifera*. Then were added FeCl3 (20 μM, Sigma-Aldrich, St. Louis, MO, USA) and ascorbic acid (100 μM, Sigma-Aldrich) to obtain the Fenton reaction . At the end of incubation, the mixture was added to 4 mL reaction mixture consisting of 36 mM thiobarbituric acid (Sigma-Aldrich) solubilized in 10% CH3COOH, 0.2% SDS, and pH was adjusted to 4.0 with NaOH. The mixture was heated for 60 min at 100 °C and the reaction was stopped by placing the vials in an ice bath for 10 min. After centrifugation (at 1600 g at 4 °C for 10 min) the absorbance of the supernatant was measured at 532 nm (PerkinElmer spectrometer).

Caspase-3 activity

After treatment the cells were scraped in 100 μM lysis buffer (200 mM Tris–HCl buffer, pH 7.5, containing 2 M NaCl, 20 mM EDTA, and 0.2% Triton X-100). Fifty microliters of the supernatant was incubated with 25 μM fluorogenic peptide caspase substrate rhodamine 110 bis-(N-CBZ-L-aspartyl- L-glutamyl-L-valyl-L-aspartic acid amide) (molecular probes) at 25 °C for 40 min. The amount of cleaved substrate in each sample was measured in a 96-well plate fluorescence spectrometer (PerkinElmer; excitation at 496 nm and emission at 520 nm).

Paw pressure test

The nociceptive threshold in the rat was determined with an analgesimeter (Ugo Basile, Varese, Italy) according to the method described by[63]. Briefly, a constantly increasing pressure was applied to a small area of the dorsal surface of the hind paw using a blunt conical mechanical probe. Mechanical pressure was increased until vocalization or a withdrawal reflex occurred while rats were lightly restrained. Vocalization or withdrawal reflex thresholds were expressed in grams. These limits assured a more precise determination of mechanical withdrawal threshold in experiments aimed to determine the effect of treatments. An arbitrary cut-off value of 100 g was adopted. The data were collected by an observer who was blinded to the protocol.

Von Frey Test

The animals were placed in 20 × 20 cm plexiglas boxes equipped with a metallic meshy floor, 20 cm above the bench. A habituation of 15 minutes was allowed before the test. An electronic Von Frey hair unit (Ugo Basile, Varese, Italy) was used: the withdrawal threshold was evaluated by applying force ranging from 0 to 50 grams with a 0.2 gram accuracy. Punctuate stimulus was delivered to the mid-plantar area of each anterior paw from below the meshy floor through a plastic tip and the withdrawal threshold was automatically displayed on the screen. Paw sensitivity threshold was defined as the minimum pressure required to elicit a robust and immediate withdrawal reflex of the paw. Voluntary movements associated with locomotion were not taken as a withdrawal response. Stimuli were applied on each anterior paw with an interval of 5 seconds. The measure was repeated 5 times and the final value was obtained by averaging the 5 measures[64].

Cold plate test

The animals were placed in a stainless box (12 cm × 20 cm × 10 cm) with a cold plate as floor. The temperature of the cold plate was kept constant at 4°C ± 1°C. Pain-related behaviours (i.e. lifting and licking of the hind paw) were observed and the time (s) of the first sign was recorded. The cut-off time of the latency of paw lifting or licking was set at 60 s.

Western Blott analysis

The sciatic nerve, L4-L5 DRGs and spinal cord of SD rats (day 21) were homogenized in lysis buffer containing 50 mM Tris-HCl pH 8.0, 150 mM NaCl, 1 mM EDTA, 0.5% Triton X-100, Complete Protease Inhibitor (Roche), and the homogenate was incubated on ice for 30 minutes. Then, the suspension was sonicated on ice using three 10-second bursts at high intensity with a 10-second cooling period between each burst. After centrifugation (13000 ×g for 15 minutes at 4°C) aliquots containing 20 μg total protein underwent to western blot analysis. Blots were incubated overnight at 4° with a rat anti-Nrf2 (C-20) antibody (1 : 1000; SantaCruz Biotechnology, USA). After being washed with PBS containing 0.1% Tween, the nitrocellulose membrane was incubated with goat anti-rabbit horseradish peroxidase-conjugated secondary antisera (1:5000) and left for 1 h at room temperature. Blots were then extensively washed and developed using enhanced chemiluminescence detection system (Pierce, Milan, Italy) and signal intensity (pixels/mm2) quantified (ImageJ, NIH). Exposition and developing time used was standardized for all the blots. Densitometric analysis was performed using the “ImageJ” analysis software, and results were normalized vs GAPDH expression measured by a specific antibody (1 : 1000; Cellsignal, USA) as internal control.

mRNA level analysis

The sciatic nerve, L4-L5 DRGs and spinal cord of SD rats (day 21) were collected as described above. mRNA was extracted using TRI – Reagent© (Sigma Aldrich, Milan, Italy). cDNA was obtained using the iScript cDNA Synthesis Kit® (Bio Rad, Milan, Italy) according to the manufacturer's protocol. Nrf2 (Nuclear factor (erythroid-derived 2)-like 2) mRNA (GenBank accession number: NM_031789.2) was amplified using the following rat gene specific primers: forward: 5' TGA CTC TGA CTC CGG CAT TTC 3', reverse 5' TCC ATT TCC GAG TCA CTG AAC 3'. NQO1 (NAD(P)H dehydrogenase, quinone 1) mRNA (GenBank accession number: NM_017000.3) was amplified using the following rat gene specific primers: forward: 5' TCA TTT GGG CAA GTC CAT TCC 3', reverse 5' TGA GCA ATT CCC TCC TGC CCT 3'. 18S ribosomal RNA (GenBank accession number: NR_046237.1) was considered as housekeeping gene and amplified using: forward: 5' TAC CAC ATC CAA GGA AGG CAG CA 3', reverse 5' TGG AAT TAC CGC GGC TGC TGG CA 3'. Both sequences were amplified by GoTaq® Flexi DNA Polymerase 2,500 U (Promega, Milan, Italy). The amplicons were electrophoresed in 1.8% agarose gel containing ethidium bromide. The resultant bands were then quantified by densitometry and the intensity of the signal normalized to18S, thus correcting for any possible uneven loading of RNA.

Immunohistochemistry of spinal cord glia

On day 21, SD were sacrificed, the L4/L5 segments of the spinal cord were exposed from the lumbovertebral column via laminectomy and identified by tracing the dorsal roots from their respective DRG. Quantification of the number and morphology of Iba1 immunoreactive microglia (rabbit, 1:1000; Wako Chemicals, Richmond, USA) and GFAP immunoreactive astrocytes (mouse, 1:5000; Chemicon, Temecula, USA) in the superficial dorsal horns of the spinal cord were performed in four cryostat sections (20 μm) by a previously reported method[60]. Immunoreactions were visualized by Alexa Fluor 568 (1:1000, Invitrogen, Carlsbad, USA). Cells were sampled only if the nucleus was visible within the plane of section and if cell profiles exhibited distinctly delineated borders. Negative control sections (no exposure to the primary antisera) were processed concurrently with the other sections for all immunohistochemical studies.

Images were acquired using a motorized Leica DM6000 B microscope equipped with a DFC350FX camera (Leica, Mannheim, Germany). Morphologic characteristics of microglia and astrocytes were assessed by inspection of at least 3 fields (40X 0.75NA objective) in the dorsal horn areas per section. The full-specimen thicknesses were acquired as z-stack series, deconvolved using Huygens Professional software (SVI, Hilversun, The Netherlands), and displayed using ImageJ software.

For the dorsal horns in each rat we obtained a single optical density value by averaging the 2 sides, and these values were compared to the homologous average values from the vehicle-treated animals.

Quantitative analysis of GFAP and Iba1-positive cells was performed by collecting at least 3 independent fields through a 20X 0.5NA objective. GFAP-positive cells were counted using the "cell counter" plugin of ImageJ, whereas Iba1-positive cells were quantified by means of the automatic thresholding and segmentation features of ImageJ.

Figure legend

Supplementary Figure 1. Glial profile in spinal cord. Effect of repeated treatment with *Vitis vinifera* (300 mg kg^{-1} daily p.o.) was evaluated in oxaliplatin-treated rats. On day 21, the number of Iba1-positive cells was measured in the dorsal horn of the spinal cord. Representative immunohistochemical staining is shown (scale bar = 10 mm and original magnification 20X for all images). Histogram shows quantitative analysis of cellular density. Each value represents the mean ± s.e.m. ofm10 rats per group, performed in 2 different experimental sets carried out by experimenters blinded to the treatments.

References for Supplementary material

50. Di Cesare Mannelli, L. *et al.* Glial role in oxaliplatin-induced neuropathic pain. *Exp. Neurol.* **261**, 22-33 (2014).

63. Leighton, G.E., Rodriguez, R.E., Hill, R.G., Hughes, J. 1988. k-Opioid agonist produce antinociception after i.v. and i.c.v. but not intrathecal administration in the rat. *Br. J. Pharmacol.* **93**, 553–560 (1988).

64. Sakurai, M. *et al.* Oxaliplatin induced neuropathy in the rat: involvement of oxalate in cold hyperalgesia but not mechanical allodynia. *Pain*. **147**, 165–174 (2009).

Supplementary Figure 1

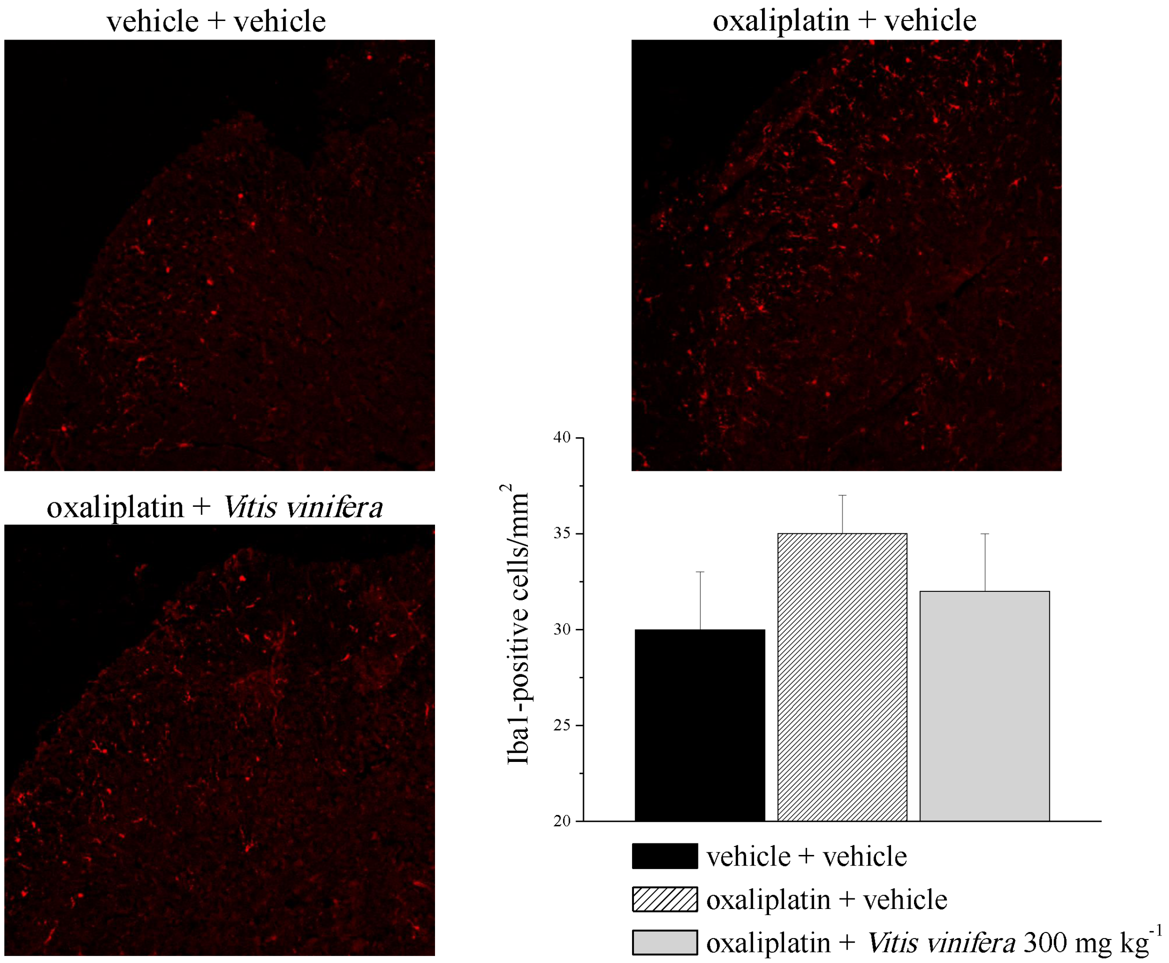

Supplement: Supplementary file 1 — Supplementary material [file 41598_2018_32691_MOESM1_ESM.pdf]
